# Enhanced prediction of cement raw meal oxides by near-infrared spectroscopy using machine learning combined with chemometric techniques

**DOI:** 10.3389/fchem.2024.1398984

**Published:** 2024-06-03

**Authors:** Yongzhen Zhang, Zhenfa Yang, Yina Wang, Xinting Ge, Jianfeng Zhang, Hang Xiao

**Affiliations:** ^1^ School of Information Science and Engineering, Shandong Normal University, Jinan, China; ^2^ Shandong University, Jinan, China; ^3^ Nanjing Forestry University, Nanjing, China

**Keywords:** machine Learning, near-infrared (NIR) spectroscopy, cement raw meal, oxides determination, meta-model

## Abstract

The component analysis of raw meal is critical to the quality of cement. In recent years, near-infrared (NIR) has been emerged as an innovative and efficient analytical method to determine the oxide content of cement raw meal. This study aims to utilize NIR spectroscopy combined with machine learning and chemometrics to improve the prediction of oxide content in cement raw meal. The Savitzky-Golay convolution smoothing method is applied to eliminate noise interference for the analysis of calcium carbonate ( ${CaCO}_{3}$ ), silicon dioxide ( ${SiO}_{2}$ ), aluminum oxide ( ${Al}_{2}O_{3}$ ), and ferric oxide ( ${Fe}_{2}O_{3}$ ) in cement raw materials. Different wavelength selection techniques are used to perform a comprehensive analysis of the model, comparing the performance of several wavelength selection techniques. The back-propagation neural network regression model based on particle swarm optimization algorithm was also applied to optimize the extracted and screened feature wavelengths, and the model prediction performance was checked and evaluated using $R_{p}$ and RMSE. In conclusion, the results indicate that NIR spectroscopy in combination with ML and chemometrics has great potential to effectively improve the prediction performance of oxide content in raw materials and highlight the importance of modeling and wavelength selection techniques. By enabling more accurate and efficient determination of oxide content in raw materials, NIR spectroscopy coupled with meta-modeling has the potential to revolutionize quality assurance practices in cement manufacturing.

## 1 Introduction

Cement is an indispensable building material, which is widely used in the fields including marine engineering, environmental protection, harbor construction, and machinery industry (Mohamad et al., 2022; Yang et al., 2022). However, unqualified cement products may reduce the safety performance of building structures, leading to safety accidents and property losses. To produce high-quality cement, it is necessary to ensure both the stability and suitability of raw materials during the batching process. The main components of cement raw material are calcium carbonate, silicon dioxide, aluminum oxide, and ferric oxide (Yang et al., 2020; Haruna et al., 2023). The quality of the raw material is measured by the three process parameters calculated from the content of these four oxides. Accurate determination of the oxide content of the raw material is critical for ensuring the quality of cement products.

Near-infrared spectrometry is the combination of spectroscopic technology and chemometrics, enabling rapid quantitative analysis of the components of the samples without extra consumptions (Si et al., 2021). 2020, Puneet Mishra et al. Accurately measured potassium and nitrogen content in dried pepper leaves using near-infrared spectroscopy combined with wavelength selection technique (Mishra et al., 2021); 2019, Yang ZF et al. Rapid determination of oxides in cement raw meal using near-infrared spectroscopy combined with synergistic interval partial least squares method. 2023 Suleiman A. Haruna et al. Near-infrared spectroscopy combined with multivariate calibration analysis was employed for the rapid and simultaneous quantitative determination of phenolic compounds in peanut seeds (Haruna et al., 2023). NIR spectroscopy has the advantages of rapid determination, improved prediction accuracy, and non-destructiveness, and is applicable to a wide range of sample types, making it an important analytical tool in many fields (Beć et al., 2020; Casson et al., 2020). Using near-infrared spectroscopy can quickly and accurately determine the content of calcium carbonate, silicon dioxide, aluminum oxide, and ferric oxide of cement raw materials—the aforementioned four active ingredients. This provides timely guidance on the raw material batching process, ensuring low-cost and environmentally friendly operation of the production line (Zhang et al., 2023).

In the determination of the oxide content of cement raw material samples, spectra were collected in the wavenumber range of 1000–2500 nm. The obtained spectral matrix had high dimensionality and contained a large number of wavenumber variables (3,112 in total), leading to irrelevance of some wavenumber variables to the oxide content, and overlapping and redundancy of information. This led to complexity in spectral analysis and low accuracy of quantitative calibration models. Previous research has demonstrated that constructing models based on effective wavelengths yields superior performance compared to those utilizing the full spectrum (Fu et al., 2022). Thus, wavelength selection holds considerable significance in the process of model building. To address this issue, this study compares several wavelength selection techniques to effectively improve model prediction performance, including the successive projections algorithm (SPA) (Liao et al., 2010), uninformative variable elimination (UVE) (Xiaobo et al., 2010), competitive adaptive reweighted sampling (CARS) (Feng et al., 2014; Kumar, 2021; Xing et al., 2021), intervals random frog hopping (iRF) (Zhong et al., 2013), and wavelength division multiple access (VIP) (Dowd, 1991). An integrated learning approach was employed for model building to enhance the predictive performance of the regression model. Four different regression algorithms were selected: Bayesian-based regression prediction model, regression prediction model based on LSBoost algorithm of integrated model, data regression prediction model based on Random Forest algorithm, and prediction model based on linear regression algorithm. In order to effectively combine the benefits of these algorithms, we constructed a particle swarm optimization (PSO)-based neural network as a meta-learner using their outputs as inputs (Gad, 2022; Abderrahim et al., 2023). By introducing the meta-learner, it is expected that the system will effectively learn how to further fuse the outputs of the base learner to improve the model performance.

This study aimed to investigate the advantage of combining near-infrared spectroscopy with machine learning and chemometrics methods to improve the quantitative prediction of oxide content in raw cement. Machine learning and chemometrics algorithms were used to preprocess spectral data, extract wavelengths closely associated with four oxides. Predictive models were constructed through meta-modeling to significantly improve the accuracy of the assessment of oxide content in raw cement. This study emphasizes the significance of wavelength selection techniques and modeling approaches in enhancing the precision of NIR spectroscopic data. Utilizing these strategies helps mitigate the influence of environmental factors on NIR spectroscopic analysis results.

## 2 Materials and methods

### 2.1 Cement sample

In this study, raw cement powder samples were obtained from two sources: the Shandong Qufu Zhonglian Cement Factory and the Shandong Linyi Zhonglian Cement Factory, totaling 197 samples. The spectra of these raw material samples were collected using a near-infrared (NIR) spectroscopy detection system. The reference background was made of polytetrafluoroethylene (PTFE), with the number of scans set at 64 and the spectral resolution set at 4 ${cm}^{-1}$ . The spectral collection range was 1,000–2,500 nm. During spectral acquisition, the room temperature was maintained between 24–26°C, indoor humidity was kept between 45% and 55%, and the reference background was re-acquired after every six samples to minimize the influence of ambient temperature and humidity on the spectra.

### 2.2 Spectral data preprocessing

NIR spectrometers typically produce high-dimensional data that include noise contamination and baseline drift caused by the equipment or external factors (Houhou and Bocklitz, 2021). Constructing quantitative calibration models directly from such spectral data is computationally complex and can be time-consuming. Noise and irrelevant information can compromise the accuracy and robustness of the calibration model (Yan et al., 2022). To address this, data preprocessing to remove such interferences is crucial. In this study, the Savitzky-Golay (Xia et al., 2020) convolution smoothing method was applied to reduce noise in the spectra.

### 2.3 Models development and hyperparameters tuning

For robust model development and evaluation, we randomly split the processed data into two subsets at a 70–30 ratio. The training set consisted of 70% of the data, and the test set comprised the remaining 30%. The training set was used for calibration applying four different modeling algorithms: a Bayesian regression-based prediction model, an integrated model utilizing the LSBoost algorithm, a data regression prediction model with the Random Forest algorithm, and a linear regression algorithm. To enhance the model’s predictive performance, we employed a Particle Swarm Optimization (PSO) neural network as the optimization algorithm for hyperparameter tuning (HT). Subsequently, we assessed the fine-tuned model using an independent test set to evaluate its generalization capacity and predictive accuracy. This comprehensive testing procedure aimed to ensure the reliability and precision of the models under development. For improved data analysis and modeling performance, all computational procedures were conducted using MATLAB version 2023b.

### 2.4 Wavelength selection

The goal of feature wavenumber variable selection is to identify the optimal subset of features from the full range of wavenumber variables in the NIR spectrum to achieve specific objectives, eliminating redundant variables to enhance the model’s prediction accuracy. In this study, we applied five different wavelength selection techniques, each optimizing the model, and ultimately selected the best one.

The Competitive Adaptive Reweighted Sampling (CARS) algorithm (Hastie et al., 2007; Xu et al., 2020; He et al., 2023) is a high-performance and efficient variable selection method based on the Darwinian principle of “survival of the fittest.” The algorithm selects wavelength points with larger absolute values of regression coefficients in the model through an Adaptive Reweighted Sampling (CARS) technique, eliminates wavelength points with smaller weights, and selects the subset with the smallest RMSECV values, effectively identifying the best combination of variables. The principle of the method is to add or remove variables iteratively to optimize the performance metrics of the model. In each iteration, the performance of the model is evaluated, and the optimal subset of variables is selected based on the RMSE. Specifically, the performance of each subset of variables is evaluated using cross-validation and the optimal number of variables is selected based on the cross-validation results.

The Successive Projection Algorithm (SPA) is a linear projection method for feature selection, commonly used for dimensionality reduction of multivariate data (Yamashita and Minami, 1969; Pang et al., 2022). SPA is founded on the concept of projection in multivariate statistics and selects variables that have the largest contribution to the target variables through repeated projection operations to form a progressively increasing subset of variables. After each projection, cross-validation is used to assess the performance of the model and a decision is made based on the cross-validation results whether to continue adding variables or to stop adding variables to achieve the optimal number of variables. This ensures that the SPA algorithm selects a subset of variables that minimizes model complexity while maintaining model performance.

The main objective of the Uninformative Variable Elimination (UVE) algorithm is to exclude variables that do not provide information relevant to the target variable (Cai et al., 2008). The algorithm progressively removes variables that do not significantly contribute to the model’s performance by evaluating their informative contribution. The advantage of UVE is that it simplifies the model and reduces unnecessary features, thereby mitigating the risk of overfitting and enhancing the model’s generalization capacity. Based on cross-validation, the optimal number can be determined by progressively removing variables that contribute less to model performance and observing whether model performance improves.

The Interval Random Frog (iRF) begins with a randomly selected subset of wavelengths, and a new subset of wavelengths is generated based on the previous subset, being accepted with a certain probability. This step is looped through N iterations to select the interval subset. In each iteration, a new subset is generated based on the previous subset and the model is trained using the training set, and then the performance of the current subset is evaluated using the test set. Repeat for several iterations and select the number of subsets with the best performance based on the cross-validation results.

Variable Importance in Projection (VIP) is a technique in multivariate data analysis that assesses the importance of each input variable to the output variable, facilitating the selection of the most relevant subset of variables. Based on cross-validation, the VIP algorithm was used on the training set to assess the importance of the variables and to rank or filter the variables. The model performance is evaluated using the test set and the process is repeated several times to ensure the stability of the results. Finally, the number of variables with the best performance is selected based on the cross-validation results.

### 2.5 Mate-modeling

**FIGURE 1:**
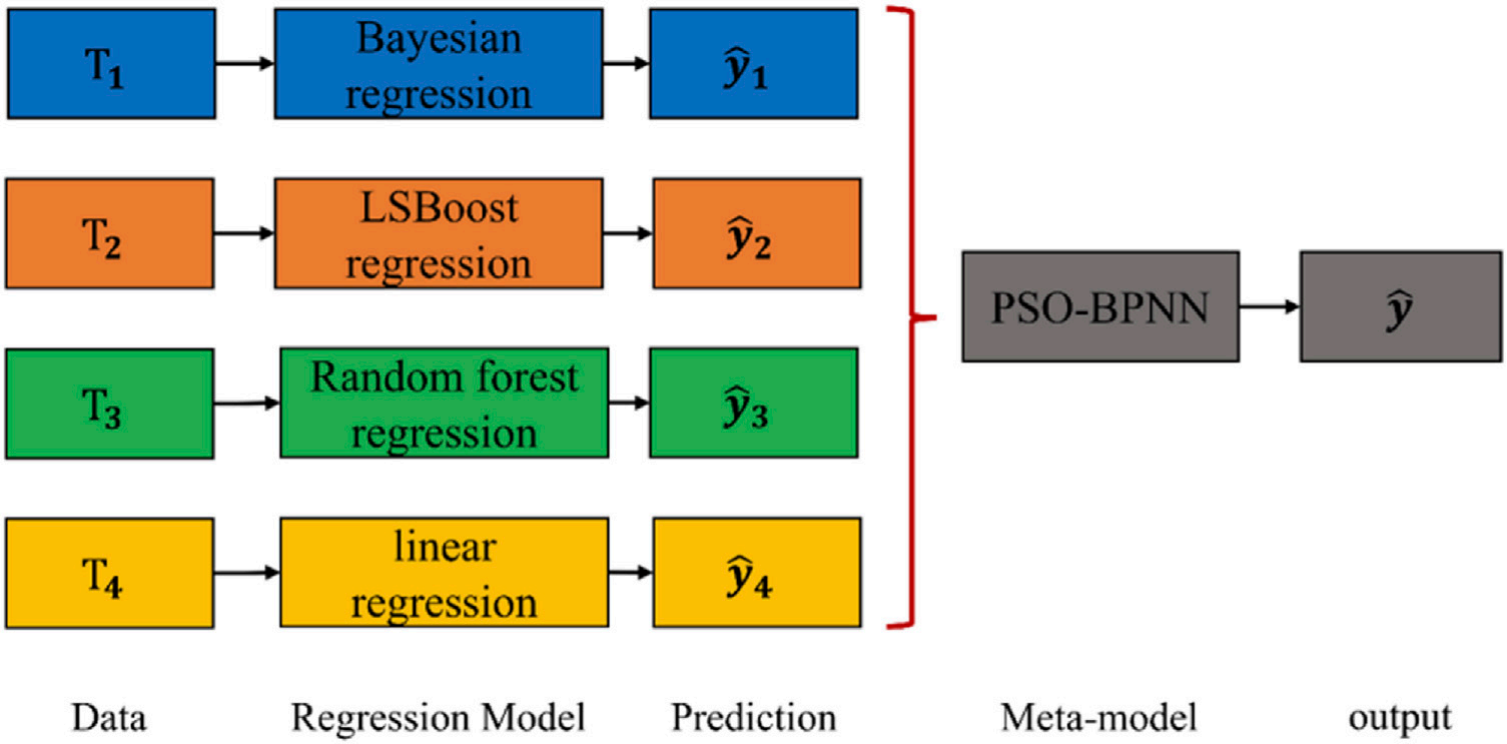
Illustration of stacking framework.

Meta-modeling (MM) is a powerful Machine Learning (ML) strategy that generates high-performance, stable, and robust models by combining multiple weakly calibrated predictions. This process is illustrated in Figure 1 and can be summarized as follows:

Firstly, four different modeling approaches were used to construct the spectral model. These include a Bayesian-based regression prediction model (Khatibisepehr et al., 2013), a regression prediction model based on the LSBoost algorithm (Friedman, 2000; sabona et al., 2022), a data regression prediction model using the Random Forest algorithm (Statistics and Breiman, 2001), and a prediction model based on the linear regression algorithm (Galton, 1886). Each sub-model was constructed using data that had undergone spectral preprocessing and wavelength selection to capture diverse information comprehensively. Secondly, a new calibration, the meta-model, was created using a Particle Swarm Optimization Backpropagation Neural Network (PSO-BPNN). This meta-model aims to integrate predictions from the four sub-models by linearly combining them to improve the accuracy and predictive power of the final model.

The developed meta-model predicts the desired response by combining the predictions of the sub-models. The advantage of this approach is that it leverages complementary information from different modeling methods, leading to a more comprehensive and robust predictive model.

### 2.6 Models evaluation

Evaluating a model’s performance is crucial for ensuring the accuracy and reliability of its predictions. Different evaluation methods provide a comprehensive understanding of the model’s performance in various aspects, which guides model tuning and improvement more effectively. $R_{p}$ is commonly used to measure the linear correlation between two variables, assessing how well the model accounts for variability in the observed data. It can take values between −1 and 1. A value of $R_{p}$ closer to one indicates better model performance in explaining data variability, while a value closer to −1 suggests a lower validity of the model. RMSE measures the discrepancy between the model’s predicted values and the actual observed values and is defined as the square root of the mean sum of squares of the residuals. A smaller RMSE value signifies more accurate model predictions. Unlike $R_{p}$ , RMSE focuses on the magnitude of the specific error, providing a more intuitive reflection of the distance between model predictions and actual observations.

$$R_{p}=1-\frac{\sum_{i=1}^{N}{\left(\hat{y_{i}}-y_{i}\right)}^{2}}{\sum_{i=1}^{N}{\left({\bar{y}}_{l}-y_{i}\right)}^{2}}$$

$$RMSE=\sqrt{\frac{\sum_{i=1}^{N}{\left(y_{i}-{\hat{y}}_{i}\right)}^{2}}{N}}$$

where $y_{i}$ , $\bar{y}$ , ${\hat{y}}_{i}$ , and N represent the measured values, the average of all measured values, the predicted values, and the total number of samples, respectively

## 3 Results and discussion

### 3.1 Calibration and validation set

After preprocessing the data and applying wavelength selection techniques, we divided the 197 spectra, which span the full concentration range covered by all samples, into two subsets: a training set and a test set. The training set, comprising 158 samples, was used for model construction, while the test set, containing 39 samples, was employed to assess the developed model’s robustness. We allocated approximately four-fifths of the total samples to the training set and the remainder to the test set to ensure coverage across the oxide concentration calibration set and validate the entire range. This allocation indicates a proper sample distribution. Given that variations in cement types can pose significant challenges to the industry, our focus was on constructing an accurate and robust predictive model. The development of this model will not only facilitate a better understanding of cement composition and properties but will also serve as a robust tool for addressing practical challenges posed by changing cement varieties. The primary goal of this work is to guarantee that the model’s predictive power is sufficiently robust to handle a diverse range of cement samples, thus supporting the future advancement of the cement industry.

### 3.2 Spectral preprocessing

**FIGURE 2:**
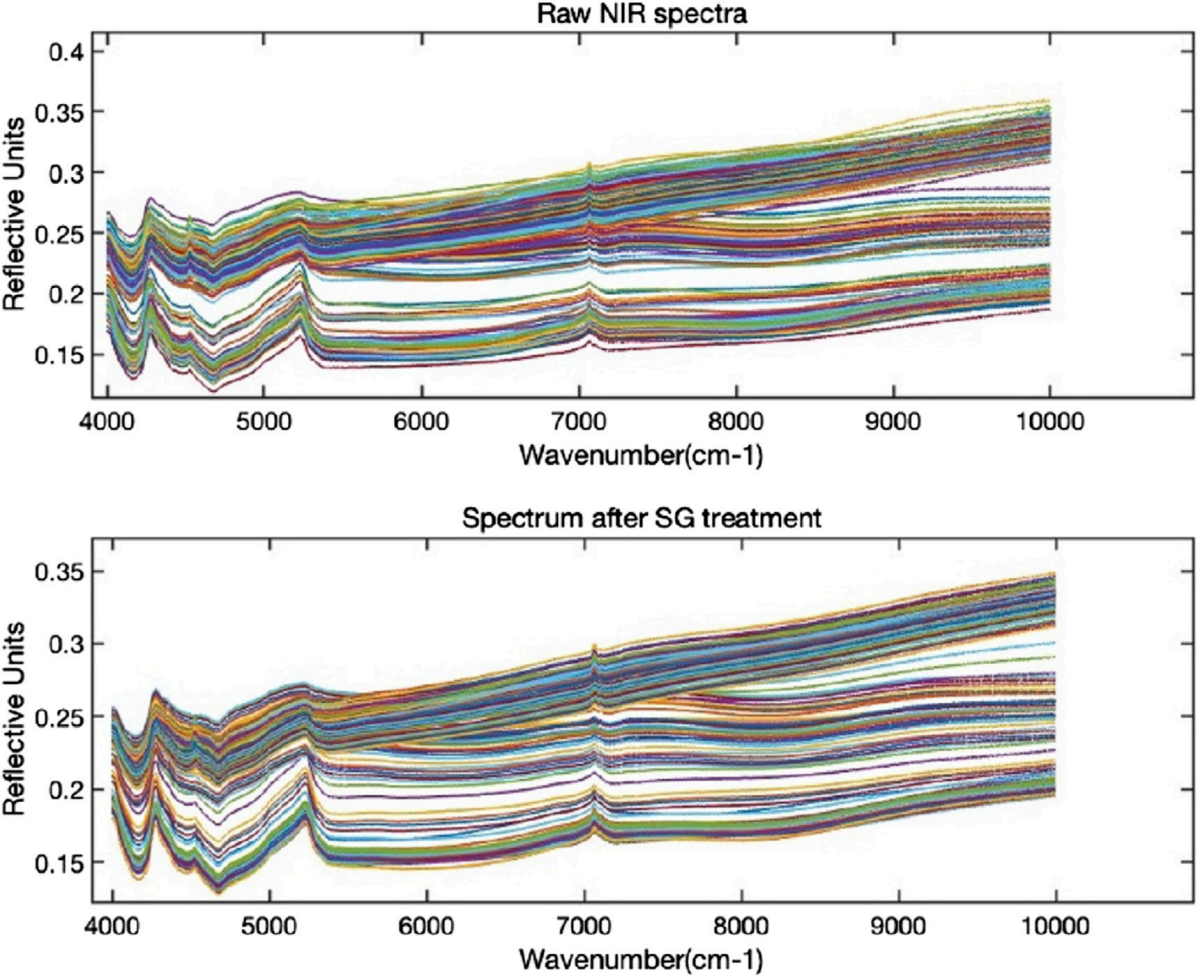
Spectral preprocessing using the Savitzky-Golay method.

The penetration depth of short-wave NIR is generally understood to be greater than that of long-wave NIR. In this study, we selected the spectral data within the wavelength range of 1,000–2,500 nm for further analysis to reduce its impact on the detection of oxide content in cement raw materials (Wang et al., 2021). As depicted in Figure 2, the spectral signals of the 197 cement raw materials may exhibit slight variations due to differences in particle size, moisture content, and particle surface state, resulting in noticeable noise. By using the Savitzky-Golay convolutional smoothing method, the smoothing and noise reduction process is performed, resulting in an improved signal-to-noise ratio.

### 3.3 Wavelength selection

Wavelength selection techniques are instrumental in reducing data dimensionality, computational complexity and enhancing model efficiency. The selected wavelengths can improve the model’s ability to perceive key features, thereby improving prediction performance. The choice of the appropriate wavelength selection technique should consider the specific application requirements and the data characteristics.

The spectrograms of the four pure oxides ( ${CaCO}_{3}$ , ${SiO}_{2}$ , ${Al}_{2}O_{3}$ and ${Fe}_{2}O_{3}$ ) showed multiple absorption peaks, a spectral feature that allowed us to analyze and evaluate the correlation between the chemical values and the spectra of the raw cement samples. Calcium carbonate samples show more obvious absorption peaks around 5,110 ${cm}^{-1}$ and 7,080 ${cm}^{-1}$ , and weak absorption peaks around 4,270 ${cm}^{-1}$ . Silicon dioxide samples show significant absorption peaks around 5,190 ${cm}^{-1}$ and 4,500 ${cm}^{-1}$ . The absorption peaks of the aluminum trioxide sample were around 5,190 ${cm}^{-1}$ and 7,200 ${cm}^{-1}$ , and also shows a weak absorption peak around 4,470 ${cm}^{-1}$ . The absorption peaks of ferric oxide are only slightly visible around 5,160 ${cm}^{-1}$ and 4,520 ${cm}^{-1}$ . Based on these observations, it can be determined that the absorption peaks near 7,200 ${cm}^{-1}$ and 5,200 ${cm}^{-1}$ are mainly caused by the vibration of H2O and Al2O3 molecules, the stronger absorption peak at 4,300 ${cm}^{-1}$ is caused by the vibration of ${CaCO}_{3}$ molecules, and the weaker absorption peak at 4,500 ${cm}^{-1}$ is caused by the superposition effect of the vibration of ${SiO}_{2}$ , ${Al}_{2}O_{3}$ and ${Fe}_{2}O_{3}$ molecules. The weak absorption peak at 4,500 ${cm}^{-1}$ is due to the superposition effect of ${SiO}_{2}$ , ${Al}_{2}O_{3}$ and ${Fe}_{2}O_{3}$ molecules (Xiao et al., 2019).

**FIGURE 3:**
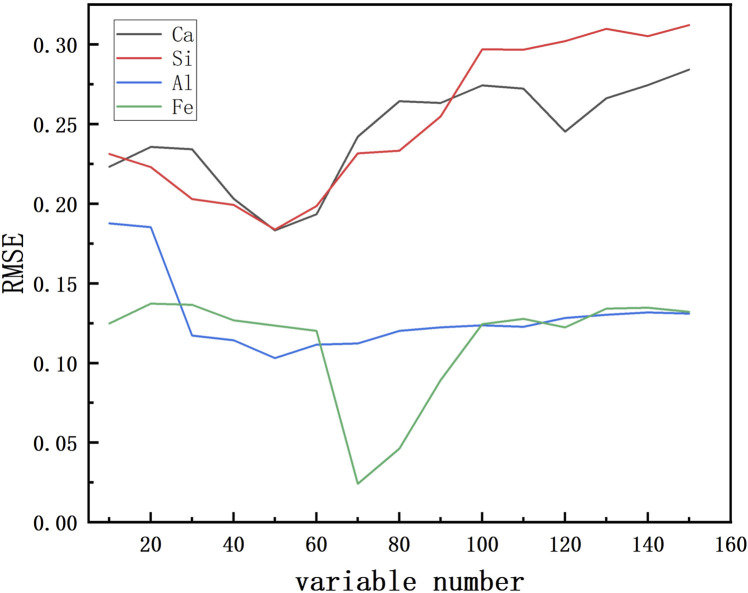
Relationship between Variable Count and RMSE.

Figure 3 shows the variation of RMSE with the increase of the number of selected variables under the competitive adaptive variable selection method. The cases of ${CaCO}_{3}$ , and ${SiO}_{2}$ are similar in that the RMSE exhibits a significant decreasing trend in the initial stage with the increase of the number of variables, indicating that the increase of the number of variables contributes to the improvement of the model’s prediction performance. But with the further increase in the number of variables, the decrease in RMSE begins to slow down and the RMSE no longer decreases significantly near the number of about 50 variables but shows a smooth or slightly increasing trend. This suggests that adding more variables near the selection of 50 variables does not significantly improve the predictive performance of the model, but instead may introduce noise or unnecessary complexity. For ${Al}_{2}O_{3}$ , the RMSE shows a decreasing trend with the increase in the number of variables and reaches a minimum at about 45 variables, after which it starts to rise smoothly. For ${Fe}_{2}O_{3}$ , on the other hand, the lowest RMSE is presented at a number of variables of about 70, indicating that the performance of the model can be significantly improved by choosing a number of variables of 70.

The application of WS techniques significantly enhances model performance, as demonstrated in Table 1. The CARS algorithm effectively reduces the number of wavelengths (NWL) from 3,112 to under 100; the IRF algorithm decreases the NWL to less than 132; the SPA algorithm to under 118; and both the UVE and VIP algorithms to fewer than 90 wavelengths. This reduction correlates with an enhancement in model prediction quality. The efficacy of various wavelength selection techniques is summarized in Table 1. Notably, CARS outperforms SPA, UVE, IRF, and VIP in predicting all four oxides in cement raw materials. CARS selects the fewest wavelengths, with counts of 47 for ${CaCO}_{3}$ and ${SiO}_{2}$ , 54 for ${Al}_{2}O_{3}$ , and 73 for ${Fe}_{2}O_{3}$ . Remarkably, ${CaCO}_{3}$ and ${SiO}_{2}$ achieve the lowest RMSEP with only 47 discrete wavelengths.

**TABLE 1:** A summary of model and several wavelength selection techniques for four dioxides. Lowest RMSEP (Root Mean Square Error of Prediction) was used to identify the best performing model. Using RPD (Relative Prediction Deviation) to assess the accuracy and reliability of spectral prediction models.

Oxides	Algorithms	WLs	$R_{P}$	RMSE	RPD
${CaCO}_{3}$	SPA	75	0.8684	0.2431	2.7348
	UVE	86	0.8035	0.2836	2.3785
	VIP	73	0.8557	0.2667	2.2324
	iRF	55	0.8420	0.2710	3.0282
	CARS	47	0.9489	0.1819	3.6482
${SiO}_{2}$	SPA	118	0.8644	0.2234	2.5256
	UVE	70	0.8631	0.2310	2.6784
	VIP	87	0.8536	0.2532	2.7633
	IRF	55	0.7044	0.2806	2.0363
	CARS	47	0.9317	0.1800	3.5327
${Al}_{2}O_{3}$	SPA	105	0.8932	0.1182	3.2472
	UVE	47	0.8730	0.1285	3.0235
	VIP	90	0.8906	0.1175	3.2036
	IRF	65	0.8212	0.1204	2.3268
	CARS	54	0.9309	0.1004	3.5242
${Fe}_{2}O_{3}$	SPA	94	0.6845	0.0788	2.2547
	UVE	83	0.6374	0.1341	2.0336
	VIP	74	0.6230	0.0764	2.3656
	IRF	132	0.6546	0.1817	2.3265
	CARS	73	0.7654	0.0228	2.9563

Following the combined SG preprocessing and CARS wavelength selection, the best model for ${CaCO}_{3}$ utilized the most informative wavelengths with Rp = 0.9489 and RMSEP = 0.1819. Similarly, for ${SiO}_{2}$ , ${Al}_{2}O_{3}$ , and ${Fe}_{2}O_{3}$ , the best models’ post-SG + CARS exhibited Rp = 0.9317, RMSEP = 0.1800; Rp = 0.9309, RMSEP = 0.1004; and Rp = 0.7654, RMSEP = 0.0228, respectively. The value of RPD is between 2.0 and 3.6 and the model has a good predictive ability. Models developed with the CARS algorithm demonstrated superior predictive performance over those using other wavelength selection techniques, indicating that CARS is more adept at selecting informative variables that enhance model accuracy.

### 3.4 The potential of meta-modeling

Meta-modeling leverages the collective strengths of multiple independent models to improve overall performance significantly (Khan et al., 2023). This integration, which may involve techniques like weighted averaging or model ensembling, endows the final meta-model with enhanced generalization capabilities. Meta-modeling achieves a balance between various aspects of the individual models by leveraging their strengths and mitigating their weaknesses, leading to more robust and comprehensive models (Qin et al., 2023). This balance is derived from an in-depth understanding of each model’s performance and characteristics. By combining multiple models, meta-modeling more effectively discerns patterns and relationships within the data, which bolsters prediction accuracy for unknown data. The goal of applying meta-modeling is to elevate predictive performance, striving for models that are both more accurate and reliable.

In this study, a novel approach was employed to determine the content of four oxides in cement raw materials. NIRS within the 1,000–2,500 nm wavelength range was explored using NIR spectroscopic detection techniques aligned with ML and chemometrics (Wang et al., 2022; Houhou and Bocklitz, 2021; da Silva Medeiros et al., 2023). This range was selected due to the presence of numerous oxide-sensitive absorption bands, especially near wavelengths 1,389, 1,600, 1926, 1956, and 2,222. The results achieved with this methodology show a high correlation with the reference method. Furthermore, the study incorporates a wavelength selection technique using the CARS algorithm, which effectively eliminates the influence of redundant and irrelevant variables (Dong et al., 2020; Wang et al., 2020), thus optimizing experimental outcomes. These techniques are instrumental in refining method accuracy by minimizing the impact of confounding variables.

Overall, the accurate and reliable determination of oxide content in cement raw meal has been successfully achieved through the integration of near-infrared NIR, ML, and chemometrics techniques. This research represents a significant advancement in the field of industrial analysis, offering an innovative method for the accurate analysis of complex substances. The successful application of this integrated technique is expected to not only enhance the monitoring of key components in industrial production but also to provide a practical and effective tool for process control and optimization. This will, in turn, contribute to improved production efficiency and quality management**.**

## 4 Discussion

### 4.1 Integration of meta-modeling approach

The introduction of a meta-modeling approach marks a significant advancement in the prediction of oxide content in cement raw meal using near-infrared (NIR) spectroscopy. By combining the outputs of four distinct modeling techniques—Bayesian regression, LSBoost regression, Random Forest regression, and linear regression—our meta-model leverages the strengths of each individual model while mitigating their weaknesses. This amalgamation of diverse modeling strategies not only enhances the accuracy and robustness of predictions but also provides a comprehensive understanding of the spectral data.

### 4.2 Advantages of meta-modeling

The meta-modeling approach offers several advantages over conventional modeling techniques. Firstly, it capitalizes on the complementary information extracted by different modeling algorithms, thereby enriching the predictive capacity of the final model. Secondly, by integrating multiple weakly calibrated predictions, the meta-model produces more stable and reliable results, reducing the risk of overfitting and improving generalization to unseen data. Additionally, the meta-model is adaptable to various datasets and can accommodate different modeling assumptions and data characteristics.

### 4.3 Comparison with previous research (SPORT method)

In comparison to our previous work on the Sequential Preprocessing through Orthogonalization (SPORT) method, which focused on preprocessing techniques to enhance prediction accuracy, the current study represents a paradigm shift towards a meta-modeling framework. While SPORT demonstrated commendable performance in minimizing scattering effects and enhancing preprocessing selectivity, its scope was limited to data preprocessing. In contrast, the meta-modeling approach presented here extends beyond preprocessing to integrate outputs from multiple modeling techniques, offering a more comprehensive solution to improve predictive performance.

### 4.4 Implications for cement industry and beyond

The findings of this study hold significant implications for the cement industry, particularly in the realm of quality control and process optimization. By enabling more accurate and efficient determination of oxide content in raw materials, NIR spectroscopy coupled with meta-modeling has the potential to revolutionize quality assurance practices in cement manufacturing. Moreover, the methodological framework developed in this research can be extrapolated to other materials analysis fields, opening new avenues for nondestructive techniques in various scientific disciplines.

### 4.5 Future directions

While the current study represents a significant advancement in predictive modeling for oxide content in cement raw meal, several avenues for future research warrant exploration. Firstly, further refinement of the meta-model architecture and exploration of alternative modeling algorithms could enhance predictive performance. Additionally, investigating the applicability of the meta-modeling approach to other spectroscopic techniques and industrial processes would broaden its utility and relevance. Furthermore, longitudinal studies tracking the performance of the meta-model in real-world cement production settings would provide valuable insights into its practical efficacy and scalability.

## 5 Conclusion

This study integrates ML, chemometrics, and NIRS for the rapid determination of four major oxides (calcium oxide, silicon dioxide, alumina, and ferric oxide) in raw cement powder. A comparison of five different WS techniques provides experimental evidence that competitive CARS algorithms yield optimal results. They effectively reduce the dimensionality of spectral data, facilitating the removal of noise and interferences irrelevant to the modeling process. This enhances model efficiency and reduces the computational load. Additionally, meta-modeling introduces a robust framework that successfully reduces the risk of overfitting and improves the model’s generalization to unseen data by integrating predictions from multiple models. This integrated approach significantly boosts the overall performance of the models, thereby increasing the accuracy and reliability of oxide content assessment in cement raw meal. By leveraging the strengths of individual sub-models, meta-modeling delivers a more dependable and robust solution for complex analytical tasks and varied data distributions.

## Data Availability

The datasets presented in this study can be found in online repositories. The names of the repository/repositories and accession number(s) can be found below: http://doi.org/10.6084/m9.figshare.25375288.
